# Discovery of reproductive tissue-associated bacteria and the modes of microbiota acquisition in male honey bees (drones)

**DOI:** 10.1128/msphere.00705-24

**Published:** 2024-12-19

**Authors:** Alexis Burks, Patrick Gallagher, Kasie Raymann

**Affiliations:** 1 Department of Biology, University of North Carolina, Greensboro, North Carolina, USA; 2 Department of Plant and Microbial Biology, North Carolina State University, Raleigh, North Carolina, USA; Third Institute of Oceanography Ministry of Natural Resources, Xiamen, China

**Keywords:** honey bee, drones, microbiota, reproductive organs, gut, acquisition

## Abstract

Honey bees are the third most economically important agricultural animal in the world due to their role as pollinators. Honey bee pollination services and all hive duties are performed by female workers, while the male drones have one job to mate and share their genetics with a virgin queen from another colony. Thus, drone fitness is directly tied to queen success and colony survival, yet they have been severely understudied compared to their female counterparts. In other insects, microbes discovered in the gut and reproductive organs have been shown to be important for reproductive success and/or overall host health. To our knowledge, the existence of microbes in drone reproductive tissues has never been investigated. Moreover, our understanding of the gut microbiota of drones is severely limited, especially when compared to honey bee workers. Here, we sampled conventional drones from healthy colonies and used 16S amplicon sequencing to identify and characterize bacteria in the reproductive organs of immature and mature drones. After identifying bacteria in drone reproductive tissues, we performed a controlled experiment in which newly emerged drones were exposed to different rearing conditions in order to determine when and how they acquire their reproductive and gut microbiota. Overall, we discovered a set of core bacteria in the reproductive and gut tissues of conventionally reared drones and revealed that social interactions are important for the proper development of the drone microbiota. Determining if these bacteria play a role in drone fecundity and health should be a goal of future research efforts.

**IMPORTANCE:**

Over the last decade, annual honey bee colony loss has increased, resulting in a critical need to determine what factors contribute to honey bee and colony health. Gut microbes have been shown to play important roles in the health of the nonreproductive female honey bee workers, which make up 90% or more of a honey bee colony. However, we currently know very little about the impact of microbes on the health of male honey bees (drones), who only make up a small portion of the colony population but play a very key role in the success of future colonies by mating with virgin queens. Here, we discovered microbes within the reproductive organs of drones and illustrated that social interactions with worker bees are necessary for the proper development of the gut and reproductive tissue microbial communities in drones. Further studies are needed to determine if microbes play an important role in honey bee reproductive health and fitness.

## INTRODUCTION

The Western honey bee (*Apis mellifera*) provides pollination services worldwide, contributing to approximately one-third of the global food supply (1, 2). Over the past 12 years, beekeepers around the globe have experienced increased colony mortality rates, with reports in the United States showing losses of up to 50.8% annually (3), the cause of which is still poorly understood. This phenomenon became widely known as Colony Collapse Disorder (CCD) in 2007. Several reasons for CCD have been proposed, such as pathogens, parasites, poor nutrition availability, climate change, and agricultural chemicals, but queen health issues have been identified as one of the leading causes of colony mortality (4, 5). As eusocial insects, honey bees rely on a social hierarchy where a single queen is the sole reproductive unit responsible for the quality and quantity of the colony population (6). Historically, a honey bee queen’s lifespan in her original colony was 2–7 years, but now queens are being superseded (replaced) more frequently, with around 50% of colonies superseding a queen every six months (4); each time a queen is superseded it triples the chance of colony mortality (4). Female honey bee workers make up 90% or more of the colony and are responsible for assessing queen health and carrying out all colony tasks including foraging, hive maintenance, and caring for the queen, drones, and newly emerged workers (7). The workers will supersede a queen for several reasons, including excess laying of unfertilized eggs (drones) due to the queen running out of or containing poor quality sperm. Male honey bees, or drones, make up only a small percentage of the colony population (<10%) during spring and summer, and their sole function is to mate with virgin queens from other colonies (8). Though drones play no direct role in their mother colony, their reproductive fitness greatly impacts the success of future queens and, thus, colonies. Despite the importance of drone health and fecundity to colony health (9), drones have been largely understudied compared to workers and queens.

**Fig 1:**
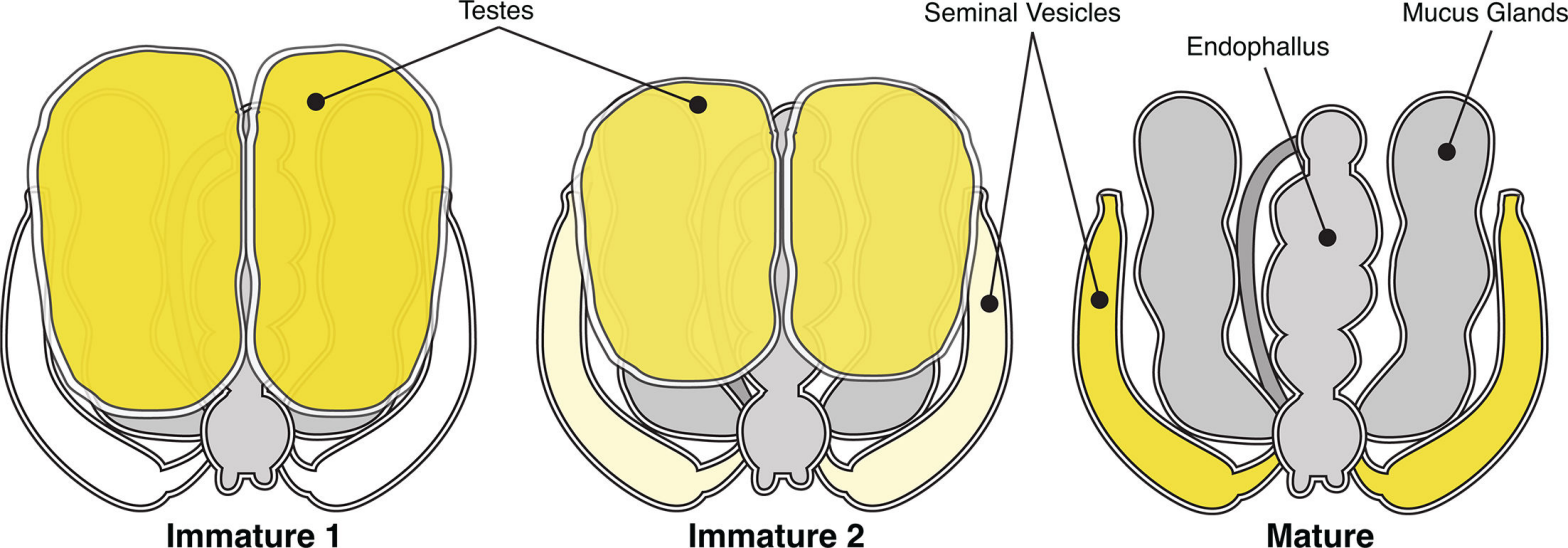
Schematic representation of drone reproductive tissues throughout development demonstrating how maturity was categorized. Immature Stage 1 (**I1**) limited to no sperm migration from testes to seminal vesicles, Immature Stage 2 (**I2**) sperm begins to migrate to the seminal vesicles, and mature (**M**) full migration of sperm from testes to seminal vesicles and necrotic or unseen testes.

Drones develop for around 24 days before they emerge as adults. During development, the entire spermatogenesis process is complete by the time a drone emerges (10). Upon adult emergence, the seminal vesicles and mucus glands are not fully developed, and all the sperm reside in the testes (Fig. 1). Six to 12 days post-emergence the sperm migrates into the seminal vesicles where it will be housed until the drone mates (11, 12). Once sperm migration is complete, the drone becomes reproductively mature and the testes are no longer functional and turn into necrotic tissue (7) (Fig. 1). Like all social hymenopteran queens, honey bee queens copulate early in life via one or few nuptial flights (13). Colony size and longevity depend on the quantity and quality of the sperm collected on these nuptial flights (14). Consequently, the seminal fluid must support high levels of sperm that can maintain viability in the male (seminal vesicles), during mating, and subsequently in the storage organ (spermatheca) of the queen over her lifetime since she will not mate again (15). It is unclear what factors affect honey bee drone reproductive fitness, but it is known that their sperm counts are highly variable (8, 11).

The gut microbiome has been shown to play a major role in honey bee worker health (16). For example, numerous studies have shown that the gut microbiota impacts immunity, pathogen defense, metabolism, and potential behavior and development in worker honey bees (17–26). The gut microbiota of workers has been extensively studied due to their large population size in the colony. It has been demonstrated that the worker microbiota is acquired through social interactions and exposure to hive materials (27, 28). Under normal conditions, the worker microbiota is thought to be fully established within 5 days post-emergence and remain relatively stable throughout a healthy bee’s lifetime (27, 29). Worker bees possess a fairly simple and highly conserved gut microbiota comprised mainly of five core taxa: *Bifidobacterium, Snodgrassella, Gilliamella, Lactobacillus*, and *Bombilactobacillus* (26, 27, 29–33). Although the core taxa present in the worker gut microbiota are conserved across individuals, several studies have revealed that strain-level diversity (within species diversity) varies across individuals, resulting in communities with various functional capabilities (34–36). It has been established that the drone gut microbiota contains some of the same taxa as workers but is overall less diverse and much more variable across individuals (37). However, to our knowledge, the modes of acquisition of the drone microbiota, including the role social interactions play in proper establishment, have not been investigated.

In recent years, microbes have been discovered in the reproductive tissues of several animals (38–42), including insects (42–46), where they have been shown to benefit host health, reproductive fitness, and offspring success (39, 43). For example, in some insects, such as mosquitoes and tsetse flies, bacteria present in reproductive tissues have been associated with host development and/or increased reproductive fitness (42). The presence of microbes in honey bee reproductive organs has not been investigated, but findings in other insects support the possibility of their existence and potential role in honey bee health and fecundity. Queen bees are difficult to study due to their indispensable role in the colony and limited experimental tractability. Because queen reproductive fitness and lifespan depend heavily on the drones that she has mated with (4), drones are a good candidate for investigating the existence and importance of bacteria in the reproductive tissues of honey bees.

In this study, we aimed to (i) determine if drones possess bacteria in their reproductive tissues, (ii) identify how gut and reproductive tissue microbiota are acquired in drones, and (iii) characterize how the microbiota communities change throughout drone maturation. To this aim, we used 16S amplicon sequencing to identify and characterize bacteria in the reproductive tissues and guts of conventional and experimentally manipulated drones. Overall, we revealed, for the first time, that drones have bacteria in their reproductive organs. Additionally, we observed that the guts and reproductive tissues of naturally emerged drones kept in a sterile lab environment without social interaction rarely contained the honey bee-associated taxa present in conventionally reared drones and workers (e.g., *Snodgrassella*, *Bifidobacterium*, *Gilliamella*, *Lactobacillus*, and *Bombilactobacillus*). Overall, we conclude that social interactions with worker bees (e.g., feeding and grooming behaviors) are critical for the acquisition and development of drone gut and reproductive microbiota. Moreover, the discovery of a conserved bacterial community in the reproductive organs of drones makes the honey bee a good candidate model system for understanding how reproductive tissue microbiota influence host fitness.

## RESULTS

### Reproductive tissue microbiota characterization

In order to determine if honey bee drones possess reproductive tissue microbiota, we randomly collected drones from four healthy hives. The drones were brought into the lab and assigned into maturity categories based on the level of sperm migration into the seminal vesicles and the degradation of the testes (Fig. 1). DNA was extracted from the reproductive tissues of each individual drone and amplicon sequencing of the 16S rRNA V4 region was used to characterize the bacteria present in 11 Immature Stage 1 (I1) testes, 14 I1 seminal vesicles and mucus glands (SV-MG), 7 Immature Stage 2 (I2) testes, 6 I2 SV-MG, and 10 Mature (M) SV-MG.

**Fig 2:**
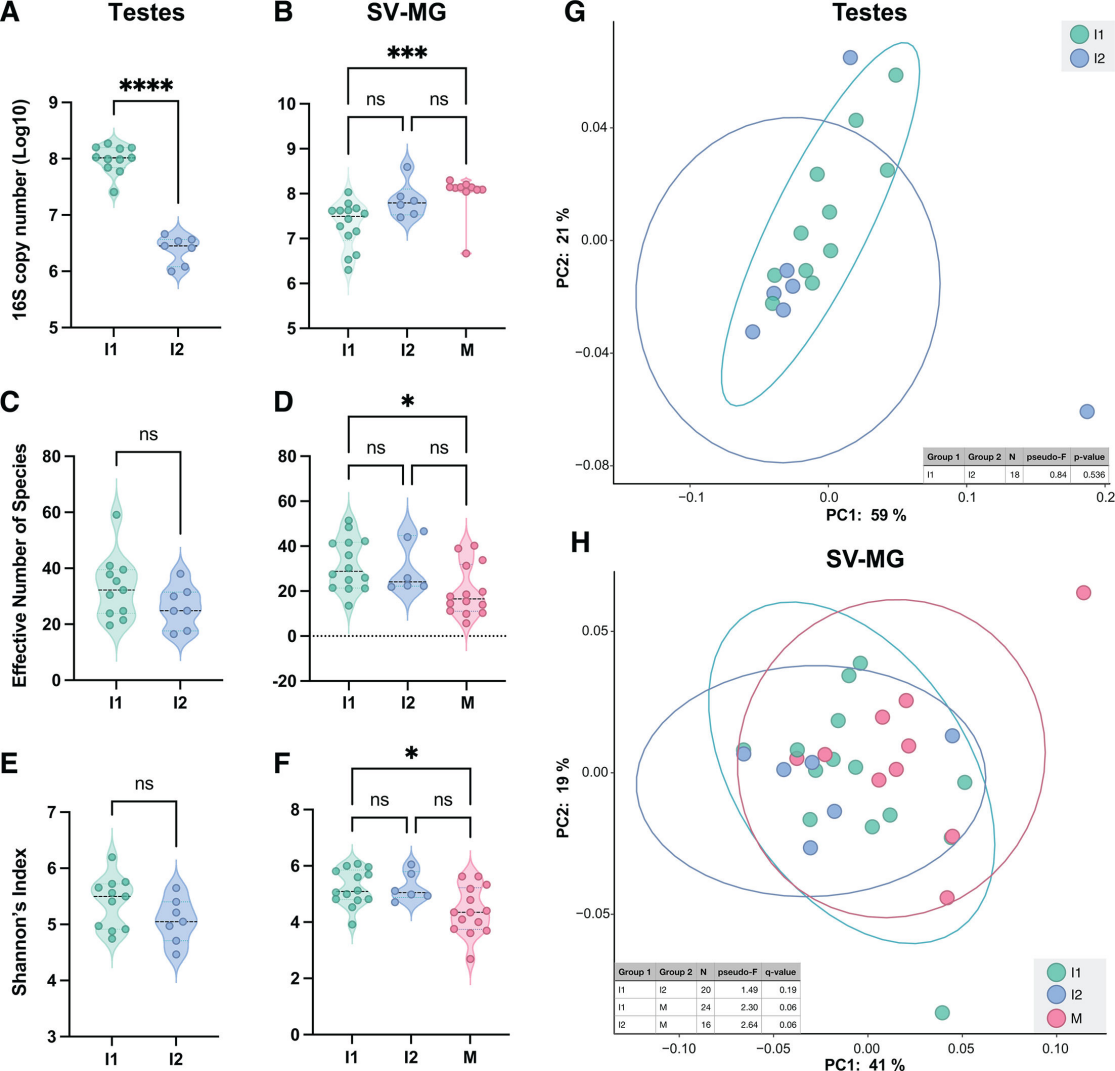
Abundance (**A and B**), alpha diversity (**C–F**), and beta diversity (**G and H**) of bacteria in the testes and SV-MG of immature and mature drones. Violin plots showing the 16S copy number (**A and B**), effective number of species (**C and D**), and Shannon’s Index (**E and F**). Principal coordinate analysis (PCoA) graphs based on weighted UniFrac (**G and H**). Abundance and alpha diversity statistical significance was determined with Mann Whitney (Testes) or Kruskal Wallis (SV-MG) with Dunn’s multiple comparison tests (**A–H**). For beta diversity comparisons, significance was tested using PERMANOVA with 999 permutations followed by Benjamini–Hochberg FDR correction (**G and H**). Ellipses represent the 95% confidence interval. Asterisks indicate statistical significance: *=*P* < 0.05, **=*P* < 0.001, ***=*P* < 0.0001, ****=*P* < 0.00001.

We first estimated the total number of bacteria present in the reproductive organs by using quantitative polymerase chain reaction (qPCR) to determine 16S copy number per tissue sample. We found that bacterial abundance significantly decreased in the testes as drones matured (Fig. 2A; *P* < 0.00001, Mann Whitney Test). We also observed an increase in bacterial abundance in the SV-MG in I1 compared to M drones (Fig. 2B; *P* = 0.0008, Kruskal Wallis with Dunn’s multiple comparison tests). Conversely, the effective number of species declined in both tissue types as drones matured (Fig. 2C and D), albeit the only significant difference observed was between the SV-MG of I1 and M drones (Fig. 2D; *P* = 0.02, Kruskal Wallis with Dunn’s multiple comparison tests). We also observed a trend of decreased bacterial richness and diversity, based on Shannon’s Index, across developmental stages in both the testes and SV-MG (Fig. 2E and F), but again, only I1 and M drones significantly differed (Fig. 2F; *P* = 0.02, Kruskal Wallis with Dunn’s multiple comparison tests). Beta diversity analysis using weighted UniFrac (Fig. 2G and H) and Bray Curtis Dissimilarity (Fig. S1) revealed no significant differences in the microbiota compositions of the testes or SV-MG across maturity stages.

**Fig 3:**
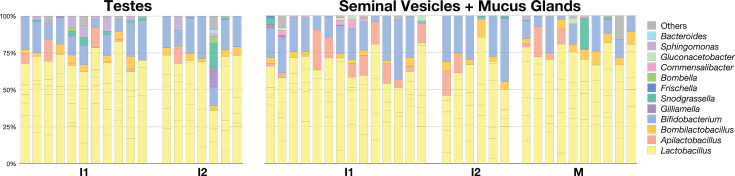
Column graph showing the relative abundance of bacterial taxa in the testes and SV-MG of drones at each developmental stage (I1, I2, and M). See Data set S1 for raw bacterial relative abundance data.

We evaluated the relative abundance of each bacterial taxon within the testes and SV-MG across maturity groups (Fig. 3; Data set S1). We found that across all developmental stages, drone reproductive organs were dominated by the *Lactobacillus* genus making up more than 50% of the community in virtually all individual testes and SV-MG, followed by *Bifidobacterium*. Notably, the *Lactobacillus* species (i.e., *L. apis*, *L. helsingborgensis*, and *L. melliventris*; see Data set S1) detected in the drone reproductive tissues are core members of the worker gut microbiota (16, 26, 27, 37). Other worker honey bee gut-associated bacteria such as *Bombilactobacillus, Snodgrassella, Gilliamella*, and *Commensalibacter* were also present in both testes and SV-MG but at low abundance (Fig. 3).

### Acquisition of gut and reproductive tissue microbiota in drones

**Fig 4:**
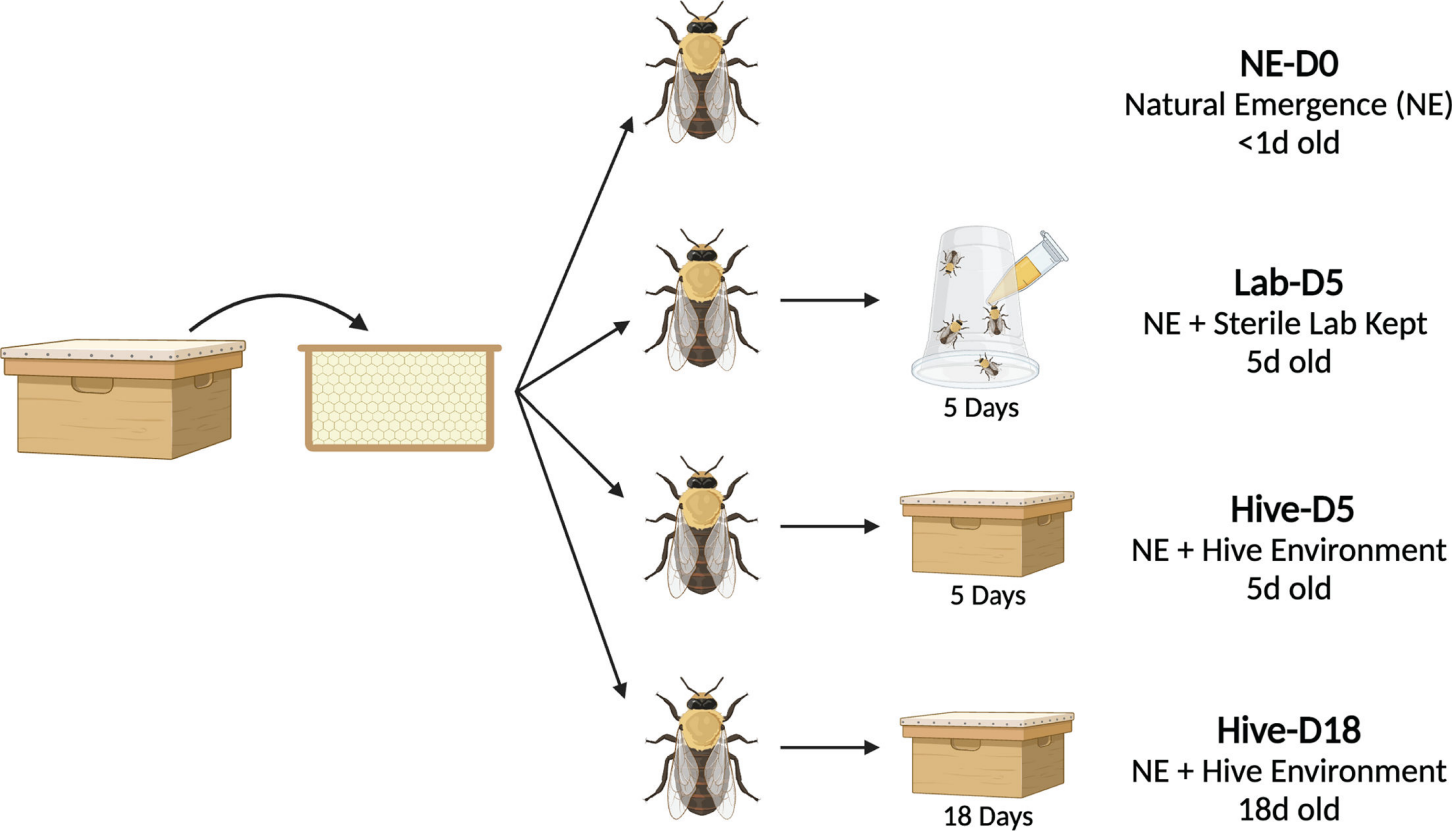
Experimental design for drone acquisition experiment. Drone brood frames were brought to the lab and naturally emerged drones were separated into four experimental groups upon emergence. NE-D0 drones were dissected the day they emerged, Lab-D5 drones were fed a sterile diet for 5 days before dissection, Hive-D5 drones were returned to the hive for 5 days before dissection, and Hive-D18 drones were returned to the hive for 18 days before dissection. Figure made with BioRender.

After confirming the presence of bacteria in the reproductive organs of drones randomly sampled from healthy hives, we aimed to investigate how drones acquire their gut and reproductive tissue microbiota. To this aim, we collected capped drone frames and allowed bees to emerge naturally overnight in the lab. We then separated the newly emerged drones into four experimental groups (Fig. 4): NE-D0, Lab-D5, Hive-D5, and Hive-D18. After gut and reproductive tissue dissection, DNA extraction, sequencing, and quality filtering, we retained 14 NE-D0, 31 Lab-D5, 28 Hive-D5, and 32 Hive-D18 guts; 22 NE-D0, 30 Lab-D5, and 30 Hive-D5 testes; 15 NE-D0, 31 Lab-D5, 32 Hive-D5, and 39 Hive-D18 SV-MG.

#### Acquisition of the gut microbiota in drones

**Fig 5:**
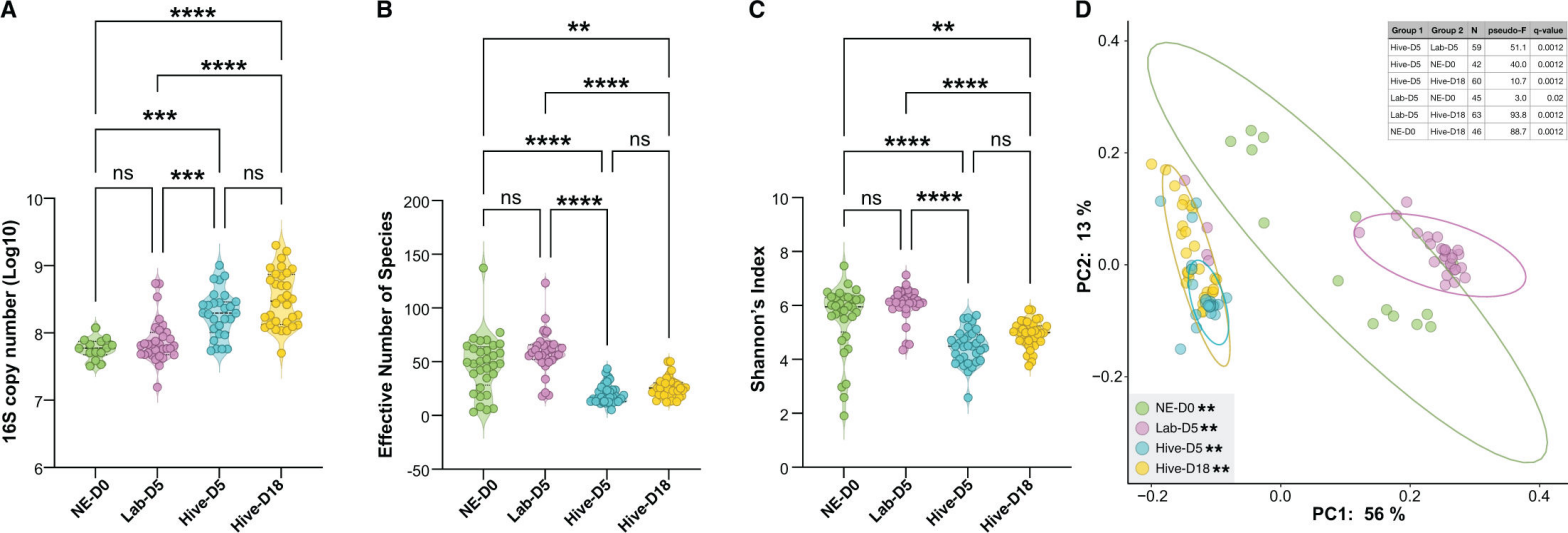
Abundance (**A**), alpha diversity (**B and C**), and beta (**D**) diversity of bacteria in the guts of drones reared under different conditions and at different developmental stages. Violin plots showing the 16S copy number (**A**), effective number of species (**B**), and Shannon’s Index (**C**). Principal coordinate analysis (PCoA) graph based on weighted UniFrac (**D**). Abundance and alpha diversity statistical significances were determined with Kruskal Wallis and Dunn’s multiple comparison tests (**A–C**). Beta diversity significance was tested using PERMANOVA with 999 permutations followed by Benjamini–Hochberg FDR correction (**D**). Ellipses represent the 95% confidence interval. Asterisks indicate statistical significance: *=*P* < 0.05, **=*P* < 0.001, ***=*P* < 0.0001, ****=*P* < 0.00001.

We estimated the absolute abundance of bacteria in the guts of drones across our experimental groups by quantifying 16S copy number in each sample. We found that the guts of drones that were returned to the hive (Hive-D5 and Hive-D18) had significantly higher bacterial loads when compared to drones with no hive interactions (Fig. 5A; *P*= <0.001, Kruskal Wallis with Dunn’s multiple comparison tests). Conversely, the effective number of bacterial species (Fig. 5B) and bacterial richness and evenness (Fig. 5C) was lower in drones that were returned to the hive than in lab-kept drones (*P* < 0.001, Kruskal Wallis with Dunn’s multiple comparison tests). Evaluation of community similarity based on weighted UniFrac (Fig. 5D) and Bray Curtis Dissimilarity (Fig. S2) demonstrated that the microbiota of drones from each experimental group all significantly differed (*P* = 0.001, PERMANOVA with Benjamini–Hochberg FDR correction). Notably, the gut community compositions of lab kept drones were very different from the hive exposed drones (Fig. 5D).

**Fig 6:**
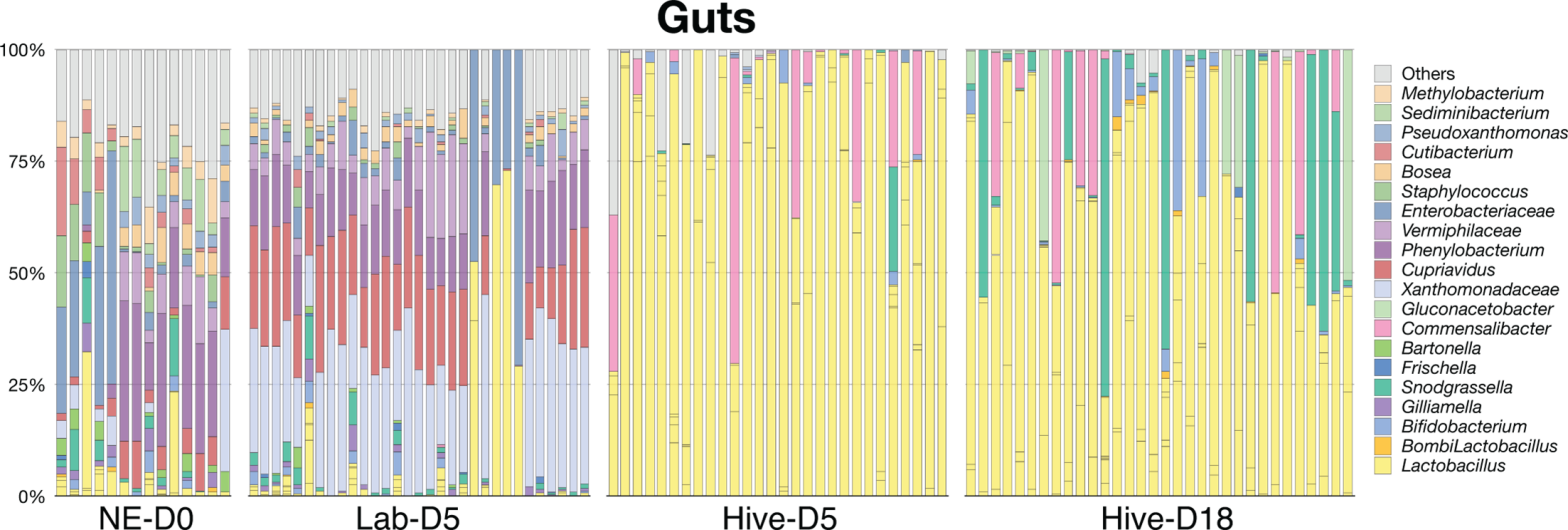
Column graph showing relative abundance of taxa in the guts of drones from each experimental group. See Data set S2 for raw bacterial relative abundance data.

To obtain a general picture of the community composition, we analyzed the relative abundance of bacterial taxa within the guts of individual drones from each experimental group (Fig. 6). We found that the lab-kept drones did not possess the typical drone gut microbiota (37) but rather contained a high abundance of plant- and soil-associated bacteria such as *Phenylobacterium, Cupriavidus,* and *Xanthromonadaceae*. The typical drone gut microbiota members (37), i.e., *Lactobacillus* and *Bifidobacterium*, only appeared and dominated in drones that were returned to the hive. Age also seemed to impact microbiota composition as Hive-D18 drones had higher relative abundances of *Bombilactobacillus*, *Snodgrassella*, *Bifidobacterium*, and *Gluconacetobacter* compared to Hive-D5 drones (Fig. S3; *P* <0.05, Kruskal Wallis and Dunn’s multiple comparison tests).

#### Acquisition of reproductive tissue microbiota in drones

**Fig 7:**
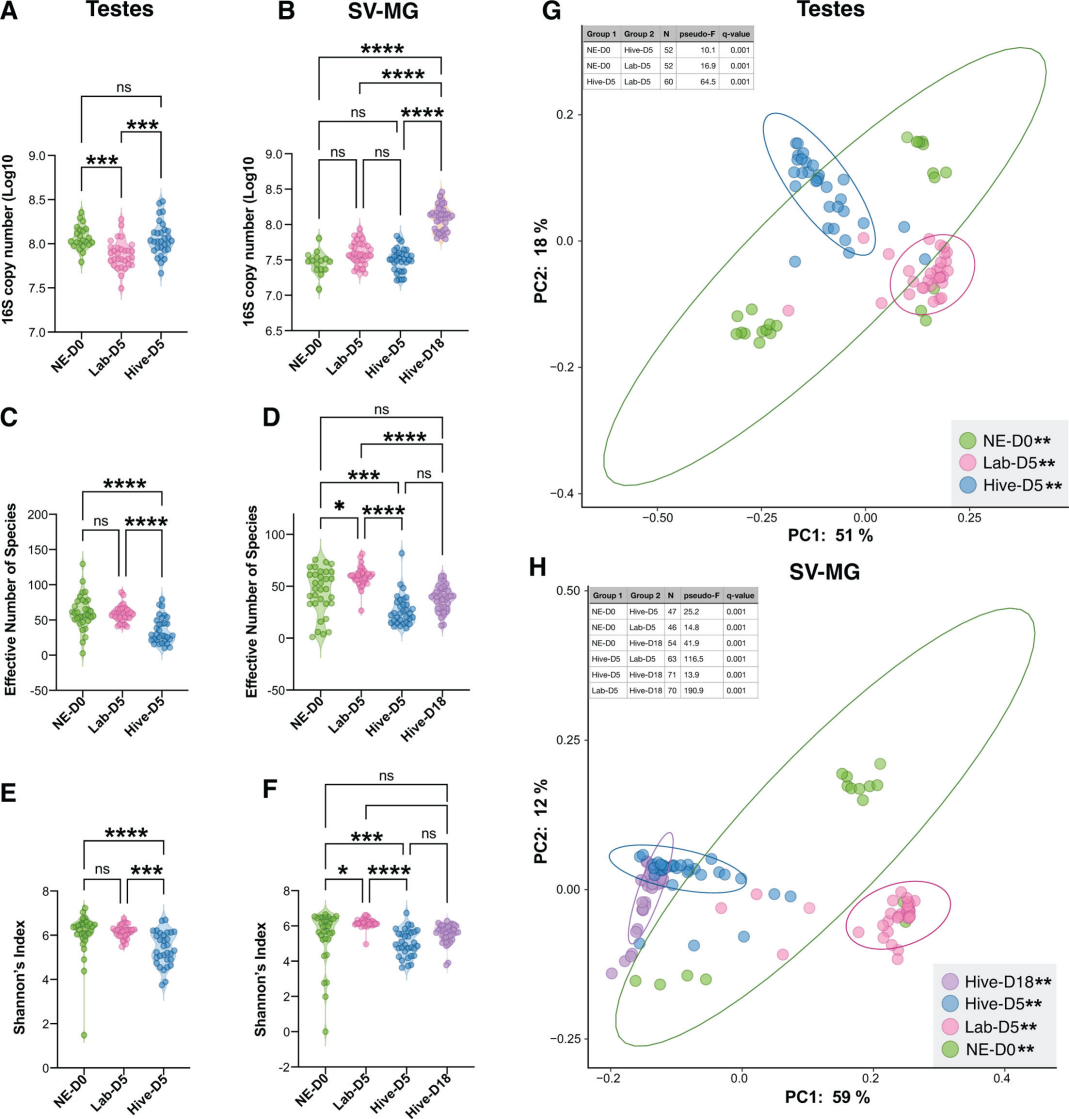
Abundance (**A and B**), alpha diversity (**C–F**), and beta diversity (**G and H**) of bacteria in the testes and SV-MG of drones reared under different conditions and at different developmental stages. Violin plots showing the average 16S copy number (**A and B**), effective number of bacterial species (**C and D**), and Shannon’s Index (**E and F**). Principal coordinate analysis (PCoA) graphs using weighted UniFrac (**G and H**). Abundance and alpha diversity statistical significance was determined with Kruskal Wallis and Dunn’s multiple comparison tests (**A–F**). Beta diversity comparisons of significance were tested using PERMANOVA with 999 permutations followed by Benjamini–Hochberg FDR correction (**G and H**). Ellipses represent the 95% confidence interval. Asterisks indicate statistical significance: *=*P* < 0.05, **=*P* < 0.001, ***=*P* < 0.0001, ****=*P* < 0.00001.

Using the same individual drones used gut microbiota analysis, we also evaluated when and how drones acquire their reproductive organ microbiota. In terms of bacterial abundance, Lab-D5 drones had significantly less bacteria in their testes compared to NE-D0 drones and Hive-D5 drones (Fig. 7A; *P* < 0.001, Kruskal Wallis with Dunn’s multiple comparison tests). Consistent with our results above (Fig. 2B), we found that bacterial abundance significantly increased with maturity, with Hive-D18 drones having higher bacterial loads in their SV-MG than NE-D0, Lab-D5, and Hive-D5 drones (Fig. 7B; *P* = 0.0001, Kruskal Wallis with Dunn’s multiple comparison tests). The effective number of bacterial species was higher in the testes and SV-MG of lab-kept drones (NE-D0 and Lab-D5) than in drones that were returned to the hive (Fig. 7C and D; *P* < 0.0001, Kruskal Wallis with Dunn’s multiple comparison tests), with the exception of NE-D0 versus Hive-D18 SV-MG (Fig. 7D; *P* = 0.36). Richness and evenness, based on Shannon’s Index, were higher in the testes and SV-MG of lab-kept drones than in Hive-D5 drones (Fig. 7E and F; *P* < 0.05, Kruskal Wallis with Dunn’s multiple comparison tests). Finally, based on weighted UniFrac (Fig. 7G and H) and Bray Curtis Dissimilarity (Fig. S4), we found that the microbial communities of both testes and SV-MG were all significantly different when compared to another treatment group (*P* = 0.001, PERMANOVA with Benjamini–Hochberg FDR correction).

**Fig 8:**
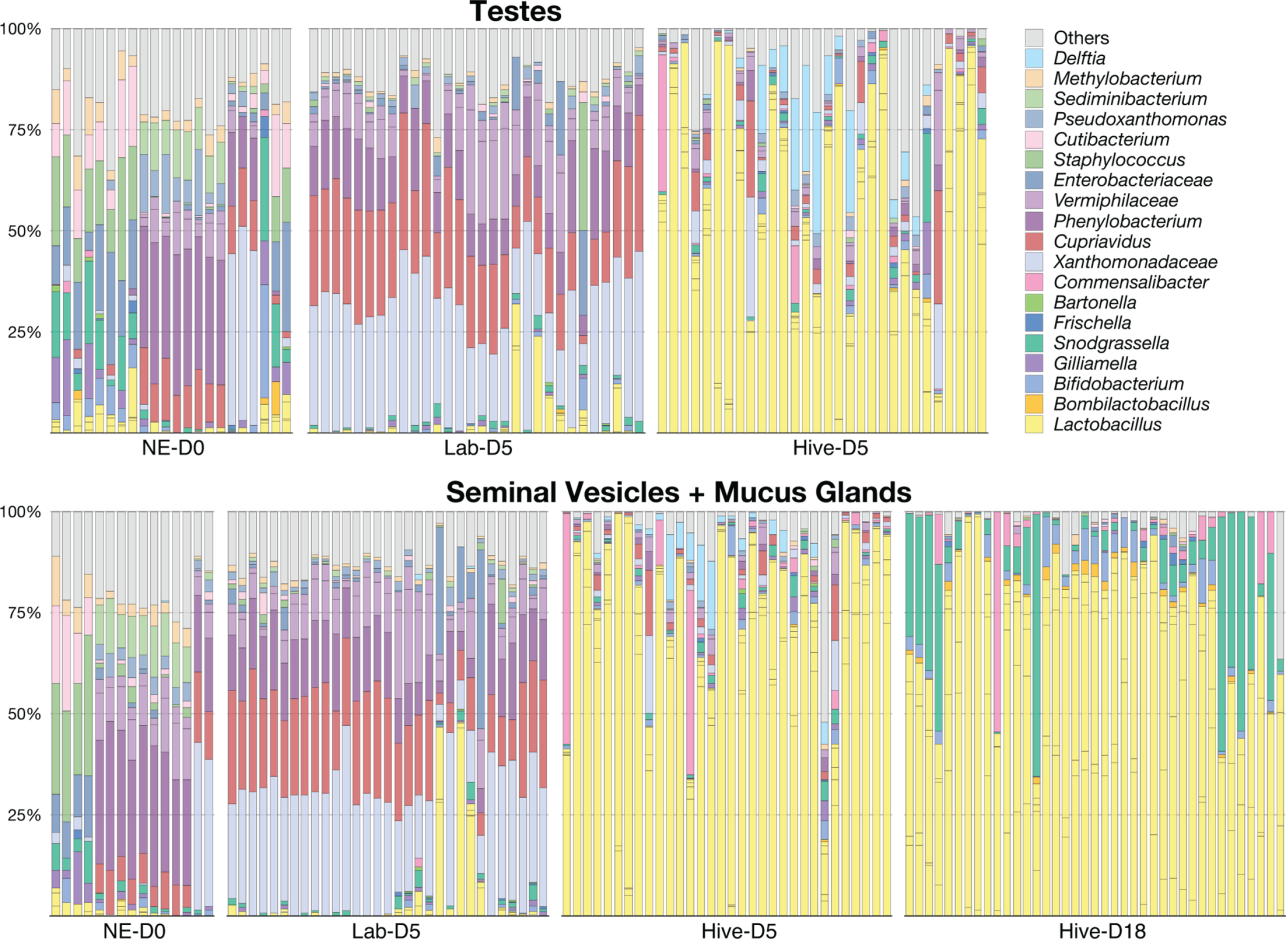
Column graph showing the relative abundance of taxa in the testes and seminal vesicles/mucus glands (SV-MG) of drones from each experimental group. See Data set S2 for raw bacterial relative abundance data.

Finally, we analyzed the taxa present in the reproductive tissues of the drones from each experimental rearing group (Fig. 8). As observed in the drones that we randomly sampled from different colonies (Fig. 3), the testes and SV-MG of drones that were returned to the hive were dominated primarily by *Lactobacillus* species (Fig. 8). Consistent with what we observed in the guts of the lab kept drones (Fig. 6), NE-D0 and Lab-D5 testes and SV-MG were dominated by bacteria that are not normally found in honey bees (Fig. 8). Microbiota composition in the SV-MG of hive-reared drones also appeared to shift with age; Hive-D18 drones had higher relative abundances of *Bombilactobacillus*, *Bifidobacterium*, and *Snodgrassella*, and a lower relative abundance of *Gilliamella* when compared to Hive-D5 drones (Fig. S5; *P* <0.05, Kruskal Wallis and Dunn’s multiple comparison tests).

## DISCUSSION

We revealed that honey bee drones possess reproductive tissue microbiota. This is a critical finding that could have major impacts on our understanding of drone reproductive fitness. We also demonstrated that proper establishment of both the gut and reproductive tissue microbiota is highly reliant on social interactions with their worker sisters, as drones allowed to naturally emerge from frames containing hive material but kept in the lab without workers did not possess “normal” microbiota. Moreover, in our experiment, the drones that were returned to the hive had limited contact with hive material as they were kept in foster cages (see Materials and Methods), indicating that it was the social contact with the workers that cared for them that promoted the establishment of their microbiota. As previously reported (37), we found that drones have less diverse gut microbial communities than workers and are mainly dominated by *Lactobacillus* species. This may be due to the fact that nurse bees are responsible for feeding and caring for drones, and when resources are low, nurses often neglect drones (47). We hypothesize that the amount of social contact a drone receives during and after emergence dictates how well they will establish their microbiota. Diet and/or lifestyle could also explain the differences between the microbiota composition of workers and drones. For example, workers consume significantly more pollen than drones (48), and the diet of worker honey bees shifts as they age and transition from nurses to foragers (49). Worker bees also spend a significant amount of time outside of the hive once they become foragers, whereas drones only leave the colony to partake in mating-related flights (50).

We found a surprisingly high number of bacteria (based on 16S copy number) within gut and reproductive tissues of lab-kept drones (NE-D0 and Lab-D5), albeit these communities did not contain the typical honey bee-associated taxa. One explanation for this finding could be the lack of exposure to native bacteria, which would normally outcompete environmental microbes and prevent them from “taking over.” In the normal hive environment, drones are assisted with removing the wax capping over their cells during emergence and are fed immediately by nurse worker bees, presumably starting the acquisition of their microbiota. Thus, we predict that all or most of the bacteria present in our lab-reared drones originated from them chewing their way out of the capping of their cells, which contains mostly environmental microbes and is composed of plant materials. It is important to note that a large portion of the NE-D0 samples did not make it through sequencing, likely due to the absence of bacteria in both their gut and reproductive tissues. Moreover, during quality filtering, we identified over 10% of the reads from the guts of lab kept drones were assigned to mitochondria, chloroplast, or were unassigned, and for some individuals, these reads accounted for upward of 40% of the total reads (Fig. S6), suggesting very few bacteria were present. Both chloroplast and mitochondrial reads are sometimes detected in 16S surveys of the honey bee gut, but at very low abundance (e.g., <1%) due to the presence of the host tissue and plant material. Additionally, we detected an abnormal percentage of the 16S amplicon reads assigned as mitochondria (or unassigned) from the testes of lab kept drones, particularly in the NE-D0 testes. In immature drones, the testes are filled with sperm, and in honey bees, mitochondria make up a majority of the sperm midpiece (51). Therefore, the 16S copy numbers determined by qPCR in lab kept drone tissues could have been overestimated due to off-target amplification of mitochondria and/or chloroplasts rather than actual amplification of bacterial 16S copies. In fact, after completing this study, we realized that the 16S primers we used for qPCR are particularly prone to off-target amplification of mitochondria, especially in samples with few bacteria (52).

Although the gut and reproductive tissue bacterial communities were similar between Hive-D5 and Hive-D18 drones (e.g., both predominately composed of *Lactobacillus*), we observed some differences in the absolute abundance of bacteria and the relative abundance of taxa, which may indicate that the reproductive and gut microbiota shifts as drone mature. It is important to note that we did not quantify the absolute abundance of each key species in our study; therefore, we cannot conclude that the shifts we observed based on relative abundance reflect the actual abundance of these species. Additionally, in this study, the NE-D0, Lab-D5, and Hive-D5 drones were sampled at the end of the summer of 2021 and the Hive-D18 drones were sampled in the spring of 2022 from a different colony. The difference in sampling strategy was due to the fact that in the summer of 2021 the workers completely stopped caring for the drones, so they died before reaching maturity. Thus, we had to obtain and analyze mature drones in the following spring. Although colony origin has been shown to have little impact on worker gut microbiota composition (53), a previous study has shown that season can affect the absolute abundance and composition of bacteria in the work gut (54). Specifically, winter worker bees have been shown to possess a higher absolute abundance of bacteria and lower community alpha diversity (54). Thus, it is possible that the differences observed between our Hive-D5 and Hive-D18 drones could be due to seasonal differences. However, we observed higher bacterial loads in the Hive-D18 drones that were sampled in the spring than in the Hive-D5 drones which were sampled in late summer. Moreover, we found no significant changes in alpha diversity in the gut or reproductive tissues between Hive-D5 and Hive-D18 drones and both were dominated by *Lactobacillus* species. Additional studies are needed to determine if and how seasonal changes, hive origin, and other environmental factors impact the microbiota of drones.

Although the taxa found in the gut and reproductive organs of drones were very similar, our sterilization methods (including ensuring the gut was never ruptured) and the high bacterial load detected in the reproductive tissues suggest this is not due to contamination by the gut microbiota during sampling. Close similarities between the gut and reproductive tissue microbiota communities have also been reported in other insects. For instance, in the tomato leafminer (*Tuta absoluta*) (55) and some mosquito species (43), several of the same bacterial taxa have been observed in both the gut and reproductive tissue microbiota. Unlike most insects, honey bees have highly specialized co-evolved gut communities (30), making it plausible that some of the core bacterial taxa evolved to specifically inhabit one tissue niche and that the resulting communities have functional and strain-level differences. It is mechanistically unclear how microbes colonize the reproductive tissues. Given the similarity in taxonomic composition between the gut and the reproductive tissues within individual drones, we hypothesize that the immune system and the gut lining, which is semi-permeable in adult bees (56), are not fully developed when bees emerge, allowing bacteria to colonize the gut and move to other organs, like the reproductive tissues. However, future studies investigating community organization (e.g., using FISH staining/fluorescent tagging) and strain-level diversity (e.g., via metagenomic sequencing and culturing) are needed to validate these hypotheses.

The gut microbiome of worker honey bees has been extensively studied and shown to impact worker health in a variety of ways. For instance, gut microbes play indispensable roles in metabolism, pathogen defense and immunity, and potentially proper development in workers (17–20, 25, 26). Although the impact of the gut microbiota on drone health has not been directly investigated, due to its similarity to workers, it is assumed to play similar roles in drones. As this is the first study, to our knowledge, to identify and characterize the honey bee drone reproductive tissue microbiota, we can only speculate that, as in other animals, it could have an impact on drone health and fecundity, which, in turn, could impact the queen with which he mates and, thus, the success of future colonies. While not specifically evaluated here, we hypothesize that microbes play a role in honey bee drone and queen fecundity. Future work is needed to characterize the function of the bacteria present in the reproductive tissues, but our study demonstrates that reproductive tissue-associated microbes should be considered when investigating the health and reproductive fitness of drones. Additionally, aside from being a great system for addressing fundamental questions about gut microbial communities, our results suggest honey bees could also be a good model system for reproductive microbiome research.

## MATERIALS AND METHODS

### Sterile dissections

All the bees and colonies utilized in this study were from the Raymann Research Apiary located at the Plant and Pollinator Center (PPC) on the Gateway North Research Campus in Browns Summit, North Carolina. Immediately upon collection, drones were surface sterilized by submerging them in 100% molecular grade ethanol to minimize contamination from surface bacteria. Drones were then pinned anterior side down to a dissection plate via their thorax and distal abdomen. The drone’s abdomen was opened via lateral incisions and then removed for an unobstructed posterior view of the internal abdomen. Each reproductive organ group was removed individually before the gut and tools were flame sterilized between organs and individuals. Immature drone dissections went in order of testes, seminal vesicles, and mucus glands, then the gut. Mature drone dissections ordered seminal vesicles and mucus glands, then the gut. If the gut ruptured during dissection, the individual was thrown out. Dissection methods were adapted from Carreck et al. (57). Samples were frozen at −80°C and then allowed to thaw at room temperature before the DNA extraction began.

### Reproductive tissue microbiota characterization

A total of 33 healthy drones were selected at random from four healthy hives, which had not been treated with any hive medications (e.g., antibiotics or miticides). Reproductive organs, testes (only in immature drones), and seminal vesicles/mucus glands (SV-MG) were sterilely dissected without gut rupture. The maturity level of the drones was based on sperm migration/semen location into three categories: (i) migration had not started and all semen was still in the testes [Immature 1; I1], (ii) migration had started, but the majority of semen was still located in the testes [Immature 2; I2], and (iii) migration was complete into the seminal vesicles and the testes were shriveled or unfindable [Mature; M]. Drone semen is tan in color and easy to visualize within the transparent testes and/or seminal vesicles (Fig. 1). A total of 19 drones were categorized as I1, 9 as I2, and only 5 as M, a total of 33 successful dissections. Due to only 5 of 33 randomly selected drones being reproductively mature, we included 10 more seminal vesicles/mucus glands samples from age-controlled 18-day old control drones from another experiment (not published). Eighteen-day-old drones are used for peak reproductive potential/maturity (11). This addition resulted in 15 mature seminal vesicles/mucus gland samples for the M group. Tissues were kept at −80°C until further analysis.

### Acquisition of gut and reproductive tissue microbiota in drones

A queen from a healthy hive was trapped on one side of a frame made of drone-sized cells using a push-in cage only allowing workers to pass through for 24 h to induce the laying of age-controlled drone eggs. After 24 h, the frame was checked for eggs, if present the queen was released (Day 0). The drone frame was returned to the hive with the push-in cage to avoid additional eggs being laid outside of the 24 h period. The frame was kept inside the hive to rear the drones naturally until Days 16–18. Prior to emergence, the frame was removed from the hive and placed in a dark incubator at 33°C and 75% RH to mimic natural hive conditions and checked every 24 h for naturally emerging drones. Any drones found on the frame were collected and either (i) sacrificed that day (NE-D0), (ii) placed into cup cages (modified from (58) for 5 days and fed sterile sugar syrup and irradiated (e-beam radiation between 15-22 kGy) pollen (Lab-D5), (3) placed in a drone foster cage and returned to the mother colony for five days (Hive-D5) or (4) placed in a drone foster cage and returned to the mother colony for 18 days (Hive-D18). Drone foster cages are small square cages placed on top of the frames inside of the hive that restricts drones and queens from passing in and out, but workers can pass through (due to their small size) to care for the drones. Five days were chosen for the Lab-D5 and Hive-D5 groups because the worker gut microbiota has been shown to fully establish 4–5 days post-emergence (27, 29). For the laboratory-reared bees, seven cup cages containing 10 bees per cup were supplied with filter sterilized 1:1 DI water and sucrose solution and irradiated pollen (e-beam radiation between 15-22 kGy). Only drones that survived all five days in the lab were dissected and used for analysis, resulting in 32 successful dissections/individuals for Lab-D5 analysis. An additional 39 drones from a different drone frame and hive were obtained and treated the same as Hive-D5 drones but they were returned to their mother colony (in foster cages) for 18 days to serve as reproductively mature samples (Hive-D18). At the end of each experiment, the guts, testes (immature drones only), and seminal vesicles/mucus glands were sterilely dissected from each individual and the sampled tissues were kept at −80°C until further analysis.

### DNA extractions and amplicon sequencing

Dissected organs were homogenized and a phenol-chloroform with bead beating extraction was performed for each individual tissue sample, this protocol is modified from Moran et al. (32). 16S rRNA PCR amplification targeting the V4 region was performed using the 515F and 806R primers containing Illumina platform-specific sequence adaptors: Hyb515F_rRNA: 5′-TCGTCGGCAGCGTCAGATGTGTATAAGAGACAGGTGYCAGCMGCCGCGGTA −3′ and Hyb806R_rRNA: 5′-GTCTCGTGGGCTCGGAGATGTGTATAAGAGACAGGGACTACHVGGGTWTCTAAT-3′. The PCR cycling conditions were as follows: 98°C for 30 s followed by 30 cycles of 98°C (10 s), 58°C (30 s), 72°C (30 s), with a final extension at 72°C for 7 m and a hold at 4°C. The PCR product was cleaned using the Axygen AxyPrep Mag PCR Clean-up Kit at 0.8 x concentration. Samples were indexed using the Illumina Nextera XT Index kit v2 sets A and D. The PCR cycling conditions for indexing were 98°C for 2 m followed by 15 cycles of 98°C (10 s), 55°C (30 s), 72°C (30 s), with a final extension at 72°C for 7 m and a hold at 4°C. The indexed product was cleaned using the AxygenTM AxyPrep Mag PCR Clean-up Kit at 0.8 x concentration, quantified with a Qubit3.0 (Life Technologies) with the Qubit dsDNA HS Assay kit, and pooled in equal concentrations for amplicon sequencing. A 30% PhiX spike-in was included in the pooling library before sequencing to increase the diversity of the run.

### Sequencing analysis

The drones randomly assigned maturity (I1, I2, and M) were sequenced on an Illumina iSeq100 in the Raymann lab with 2 × 150 paired-end sequencing. For the experimentally controlled drones (NE-D0, Lab-D5, Hive-D5, and Hive-D18), all samples were sequenced on an Illumina MiSeq at the Genomic Sciences Laboratory at North Carolina State University with 2 × 300 paired-end sequencing. Our negative DNA extraction controls did not contain enough DNA to use for sequencing and were excluded from the sequencing runs. The iSeq 150 bp paired-end reads were merged using FLASH with a minimum overlap of 5 bp and denoised in Qiime2 (v2023.7) using the DADA2 pipeline (qiime data2 denoise-single) with –*P* trunc-len 0. The MiSeq 300 bp paired-end reads were merged using the Qiime2 (v2023.7) using the DADA2 pipeline (qiime dada2 denoise-paired) with --p-trunc-len-f 210 and --p-trunc-len-r 130.

After denoising, the data were filtered to remove reads corresponding to mitochondria, chloroplast, and unassigned taxa and reads present at less than 1% frequency. All downstream analyses were performed in Qiime2 at a sampling depth of 2000 reads per sample. This sampling depth was chosen to maximize the number of samples included in the analysis while still maintaining enough reads per sample to capture the richness of the data set. Rarefying to 2000 reads per sample resulted in retention of 54 randomly sampled drone samples; 25 I1 samples (11 testes, 14 seminal vesicles/mucus glands), 13 I2 samples (seven testes, six seminal vesicles/mucus glands), and 16 M samples (16 seminal vesicles/mucus glands) successfully made it through the analysis. Rarefying to 2000 reads per sample resulted in retaining 304 experimentally controlled drone samples: 51 NE-D0 samples (14 guts, 15 SV-MG, and 22 testes), 92 Lab-D5 samples (31 guts, 31 SV-MG, and 30 testes), 90 Hive-D5 samples (28 guts, 32 SV-MG, and 30 testes), and 71 Hive-D18 samples (32 guts, and 39 SV-MG).

Qiime2 (v2023.7) was used to perform alpha (Effective Number of Species (59) and Shannon’s Index (60) and beta (weighted UniFrac (61, 62) and Bray Curtis Dissimilarity (63) diversity analyses (64). The taxonomy of the representative sequences was determined using “qiime feature-classifier classify-sklearn” (65) using a classifier trained on the SILVA 16S v138.1 (66) reference database and the BEExact v2023.01.30 (67) reference database. The relative abundance of individual taxa and alpha diversity analyses were statistically tested using either a Mann Whitney test or a Kruskal–Wallis Test with Dunn’s multiple comparisons test, and the results were plotted using GraphPad Prism v10.2.1. Beta diversity analyses were done using weighted UniFrac (61, 62) and Bray Curtis Dissimilarity and statistically analyzed using PERMANOVA (999 permutations) with Benjamini–Hochberg FDR correction. The PCoA plots with 95% confidence intervals (stat_ellipse) were generated using Qiime2R (68).

### Quantitative PCR to estimate bacterial abundance

We amplified total copies of the 16S rRNA using the primers EUB338F: 5’- ACTCCTACGGGAGGCAGCAG-3′ and EUB518R: 5’- ACTCCTACGGGAGGCAGCAG-3′ on an Applied Biosystems QuantStudio 6 Real-Time PCR system. Reactions (10 µL) were completed in triplicate using 5 µL of universal SYBR Green (Bio-Rad, Inc.), 2 µL of molecular grade water, 1 µL (each) of 3 nM primers, and 1 µL of diluted template DNA. The PCR cycle was 95°C (3 min) followed by 40 cycles of 95°C (3 s) and 60°C (20 s). A gBlocks gene fragment (Integrated DNA Technologies) was used to create a standard curve via serial dilution in triplicate (5′- GTAACGCTTGCACCCTCCGTATTACCGCGGCTGCTGGCACGGAGTTAGCCGGTGCTTATTCGTTAGATACCGTCATAATCTTCTCTAACAAAAGGAGTTTACAATCCTAAAACCTTCATCCTCCACGCGGCGTTGCTGCTTCAGGCTTTCGCCCATTGAGCAATATTCCCTACTGCTGCCTCCCGTAGGAGTCTGGACCGTGTCTCAGTT-3′). The average *C_t_* (cycle threshold) was determined for each sample. We then estimated absolute copy number by interpolating the *C*_t_ value into standard curves of known copy numbers, from 10^9^–10^3^ copies of DNA. Our efficiency coefficient was 1.02, *y*-intercept = 35.885, and slope = −3.278. Differences in abundance based on qPCR were determined using either a Mann Whitney test or a Kruskal–Wallis test with Dunn’s multiple comparisons test, and the results were plotted using GraphPad Prism v10.2.1.

## Data Availability

The raw sequencing files generated and analyzed during the current study are available in the NCBI SRA repository under BioProject PRJNA1151022. All other data generated or analyzed during this study are included in this published article and its supplemental files.

## SUPPLEMENTAL MATERIAL

Data set S1 msphere.00705-24-s0001.xlsx Individual taxon abundance.

Data set S2 msphere.00705-24-s0002.xlsx Individual taxon abundance.

Supplemental Material msphere.00705-24-s0003.pdf Supplemental figures and captions for Data sets S1 and S2.

ASM does not own the copyrights to Supplemental Material that may be linked to, or accessed through, an article. The authors have granted ASM a non-exclusive, world-wide license to publish the Supplemental Material files. Please contact the corresponding author directly for reuse.
