# Supplementary material for: Discovery of reproductive tissue-associated bacteria and the modes of microbiota acquisition in male honey bees (drones)
Source: mSphere. 2024 Dec 19;10(1):e00705-24. doi: 10.1128/msphere.00705-24 (PMC11774027; doi:10.1128/msphere.00705-24)
Supplement: Supplemental Material — Supplemental figures and captions for Data sets S1 and S2. [file msphere.00705-24-s0003.pdf]

Supplementary Material

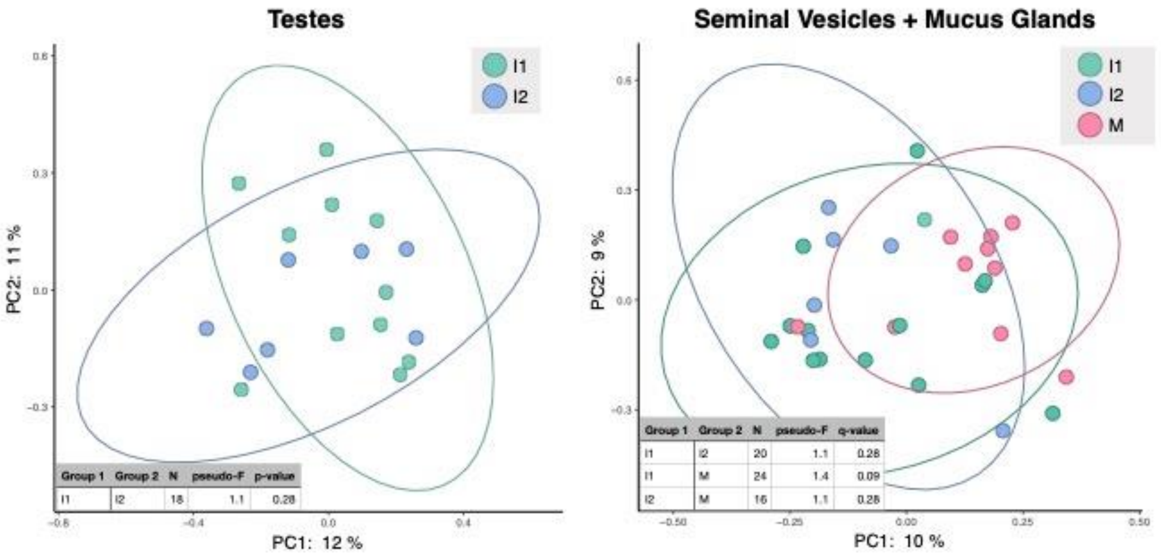

Figure S1. Principal Coordinate Analysis (PCoA) graphs based on Bray Curtis Dissimilarity. Significance was tested using PERMANOVA with 999 permutations followed by Benjamini–Hochberg FDR correction. Ellipses represent the 95% confidence interval.

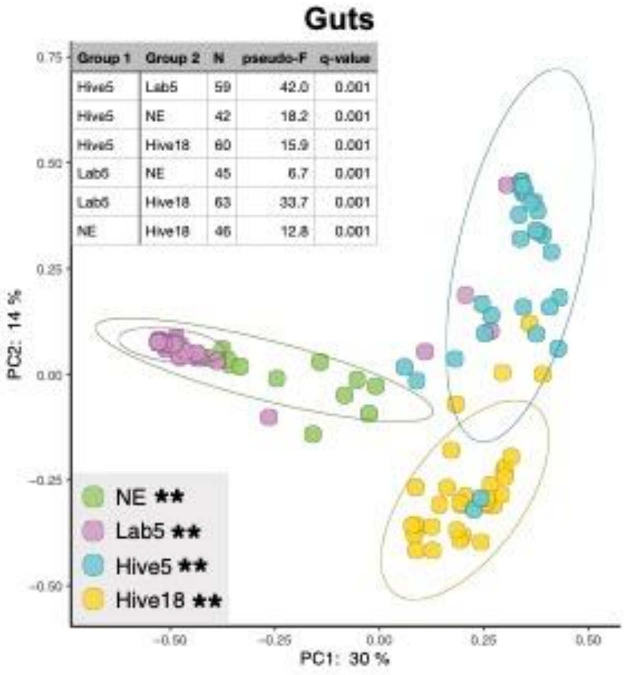

Figure S2. Principal Coordinate Analysis (PCoA) graph based on Bray Curtis Dissimilarity. Significance was tested using PERMANOVA with 999 permutations followed by Benjamini–Hochberg FDR correction. Ellipses represent the 95% confidence interval. Asterisks indicate statistical significance: **=P<0.001.

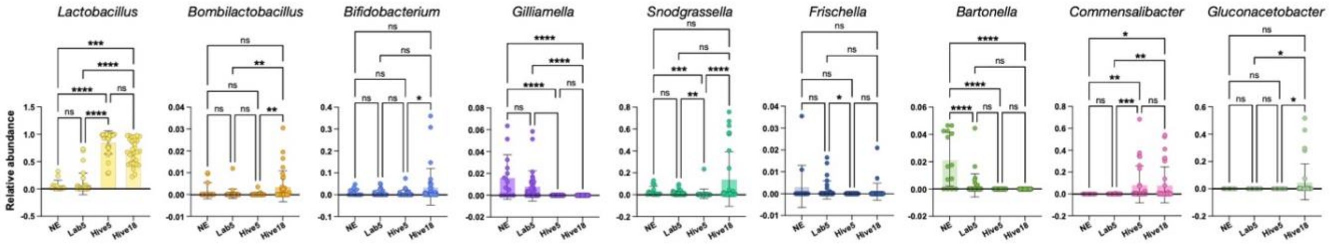

Figure S3. Comparison of the relative abundance of the nine honey bee-associated taxa detected in the guts of drones. Significance was tested using Kruskal Wallis with Dunn's multiple comparison tests. Asterisks indicate statistical significance: *=P<0.05, **=P<0.001, ***=P<0.0001, ****=P<0.00001.

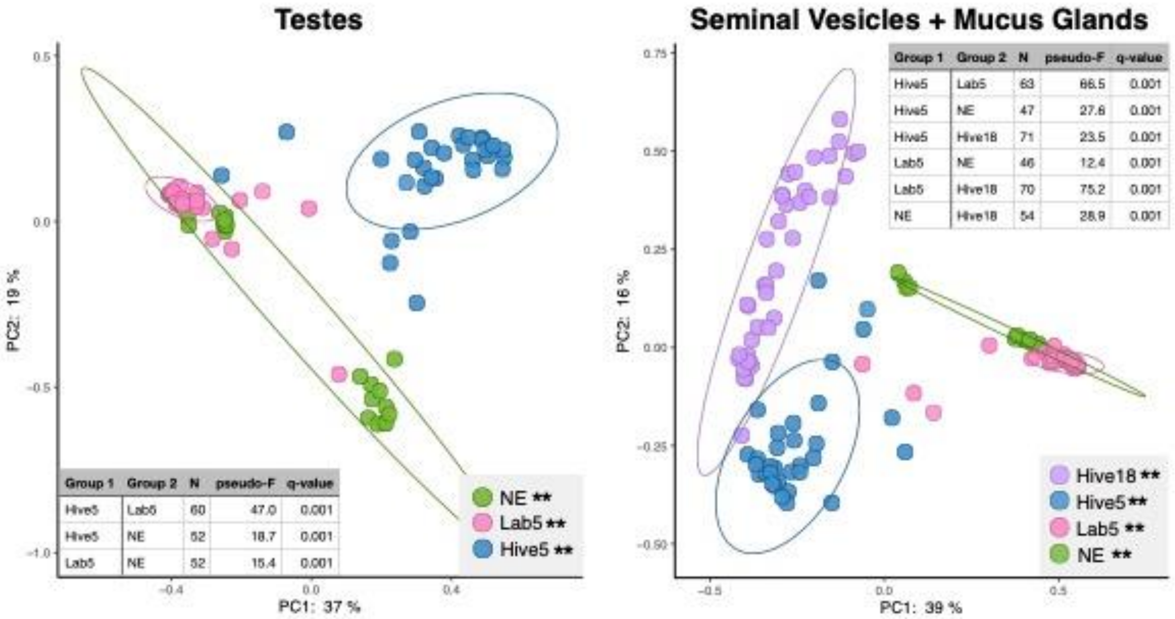

Figure S4. Principal Coordinate Analysis (PCoA) graph based on Bray Curtis Dissimilarity. Significance was tested using PERMANOVA with 999 permutations followed by Benjamini–Hochberg FDR correction. Ellipses represent the 95% confidence interval. Asterisks indicate statistical significance: **=P<0.001.

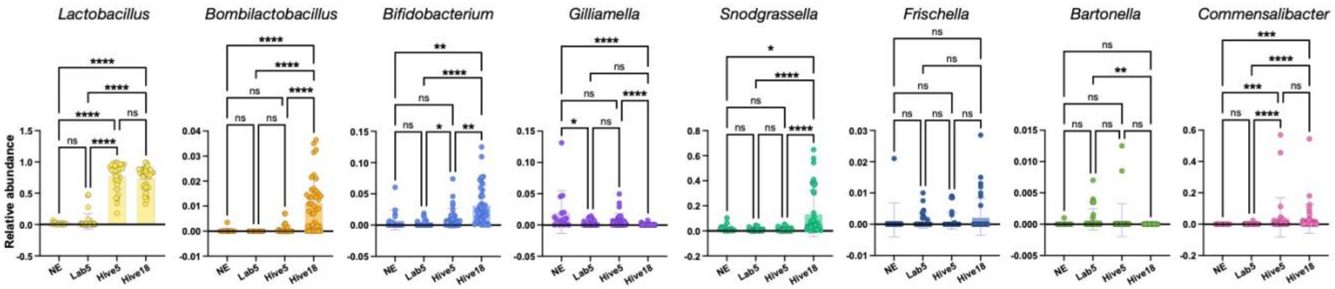

Figure S5. Comparison of the relative abundance of the eight honey bee-associated taxa detected in the SV-MG of drones. Significance was tested using Kruskal Wallis with Dunn's multiple comparison tests. Asterisks indicate statistical significance: *=P<0.05, **=P<0.001, ***=P<0.0001, ****=P<0.00001.

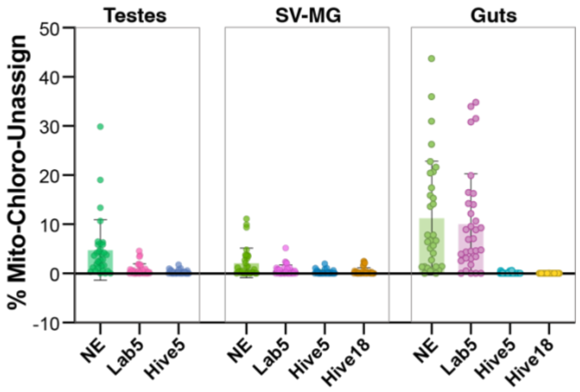

Figure S6. Percentage of sequencing reads assigned to mitochondria, chloroplasts, or unassigned in the testes, SV-MG, and guts of NE, Lab5, Hive5, and Hive18 drones. Box plots show the mean values with standard deviation and each point represents an individual bee.

Dataset S1. Relative abundance of bacterial taxa present in the testes and seminal vesicles/mucus glands of Immature 1 (I1), Immature 2 (I2), and Mature (M) drones classified to the species-level (L7).

Dataset S2. Relative abundance of bacterial taxa present in the guts, testes, and seminal vesicles/mucus glands of NE-D0, Lab-D5, Hive-D5, and Hive-D18 drones classified to the species-level (L7).
